# Supplementary material for: Mapping Rora expression in resting and activated CD4+ T cells
Source: PLoS One. 2021 May 18;16(5):e0251233. doi: 10.1371/journal.pone.0251233 (PMC8130942; doi:10.1371/journal.pone.0251233)
Supplement: S1 File — (PDF) [file pone.0251233.s001.pdf]

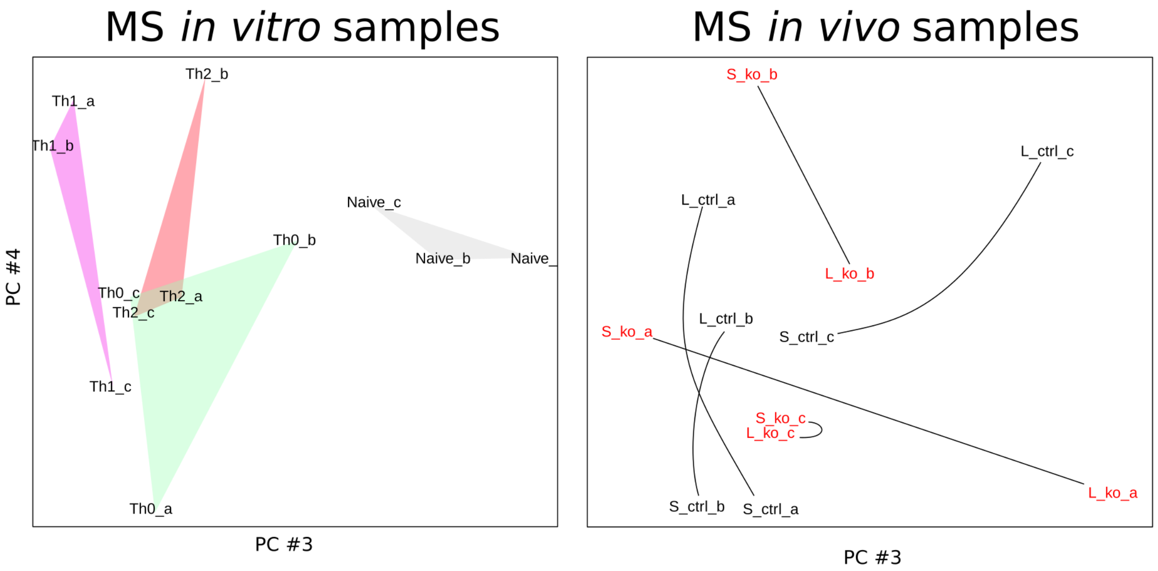

S1 Fig: PCA analysis of the LC-MS samples. *In vitro* samples separate into types. *In vivo* samples appear to primarily cluster on donors, with spleen and lung from mouse ko_c being the most clear example.

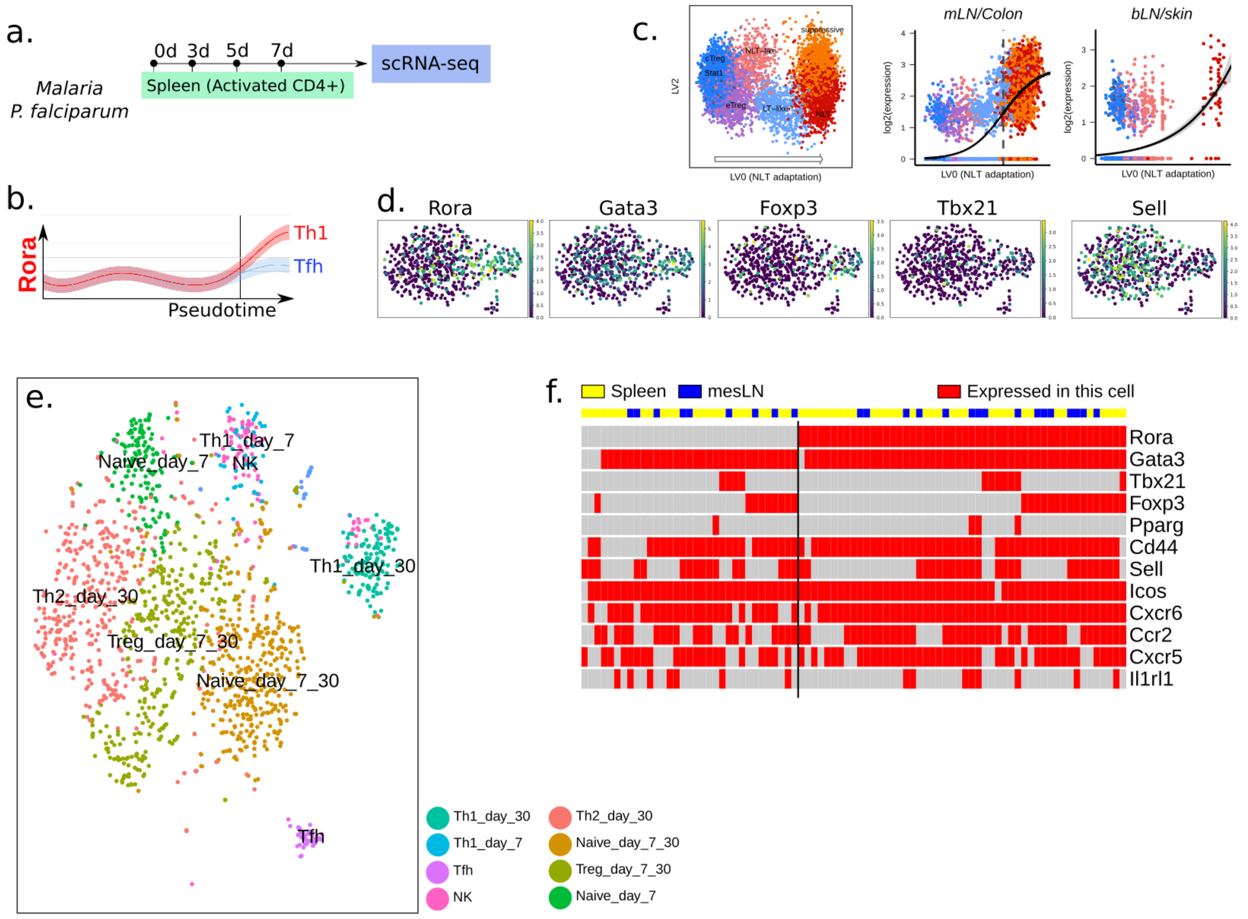

S2 Fig:

(a) Experimental design for a previous malaria *in vivo* time course scRNA-seq experiment (*1*), and **(b)** pseudo-time reconstruction of *Rora* gene expression, in Th1 and Tfh. **(c)** Panels reproduced from a study comparing Treg cells from lymphoid and non-lymphoid tissue (*2*). This study shows that Rora is mainly expressed in peripheral-like (IL10-producing) Tregs, and non-lymphoid tissue Tregs. **(d)** Expression of *Rora*, and other marker genes, in CD4 T cells in mouse melanoma (*3*) in agreement with our conclusion that *Rora* is expressed mainly in activated T cells. **(e)** Clustering of all cells related to the *N. Brasiliensis* scRNA-seq experiment. **(f)** Expression level (single cell RNA-seq) of selected genes in activated CD62L-/CD44+ T cells, 6 weeks after an *S. mansoni* infection, confirming *Rora* presence. Note that because activated CD4+ T cells were sorted for this experiment, the overlap with Rora+ and Sell- should be less than in the previous *N. brasiliensis* experiment.

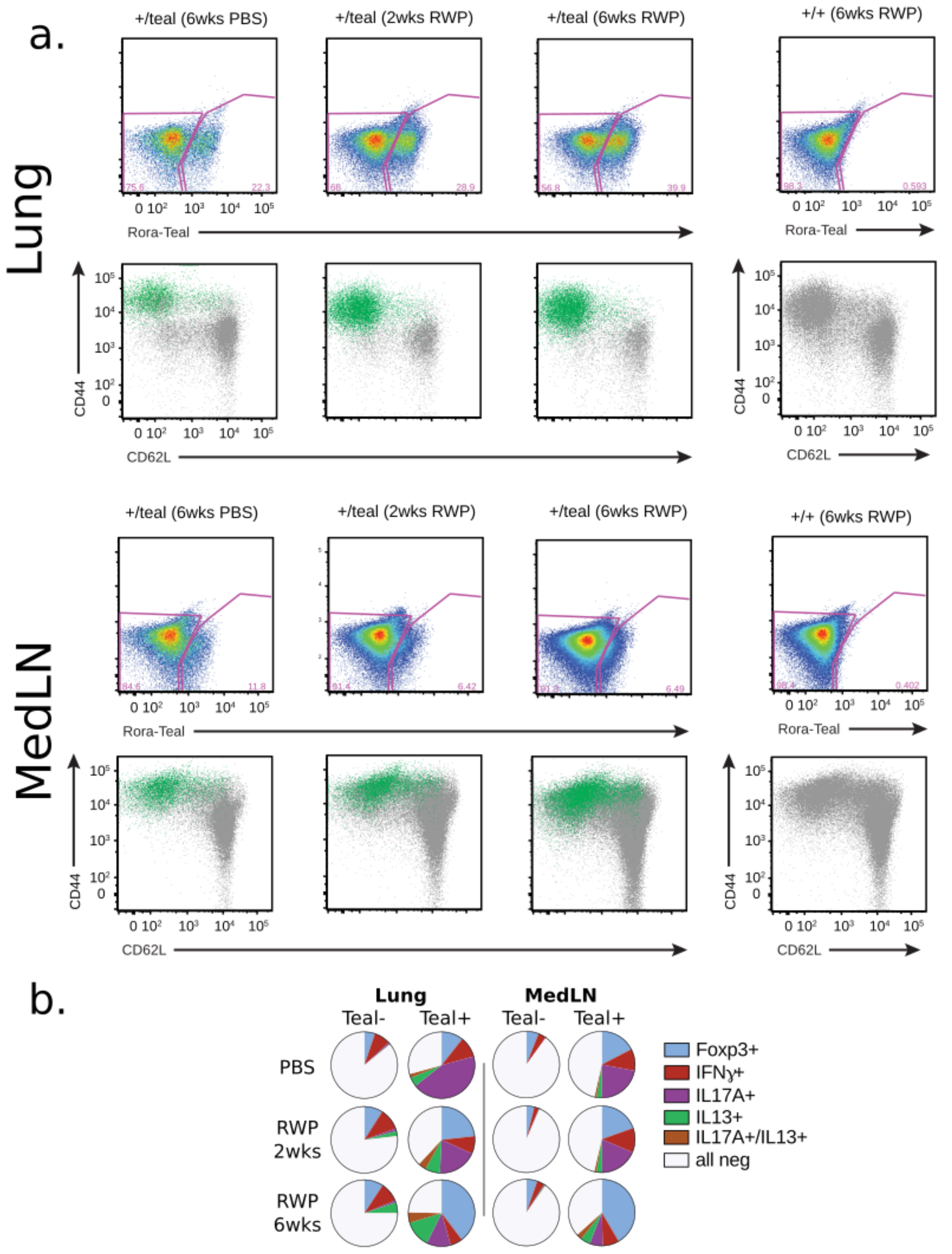

S3 Fig: (a) FACS analysis of Rora+ and Rora- CD4+ T cells from RWP treated mice.**(b)** Analogous plot to Figure 3c, except IL17 and IFN are also included.

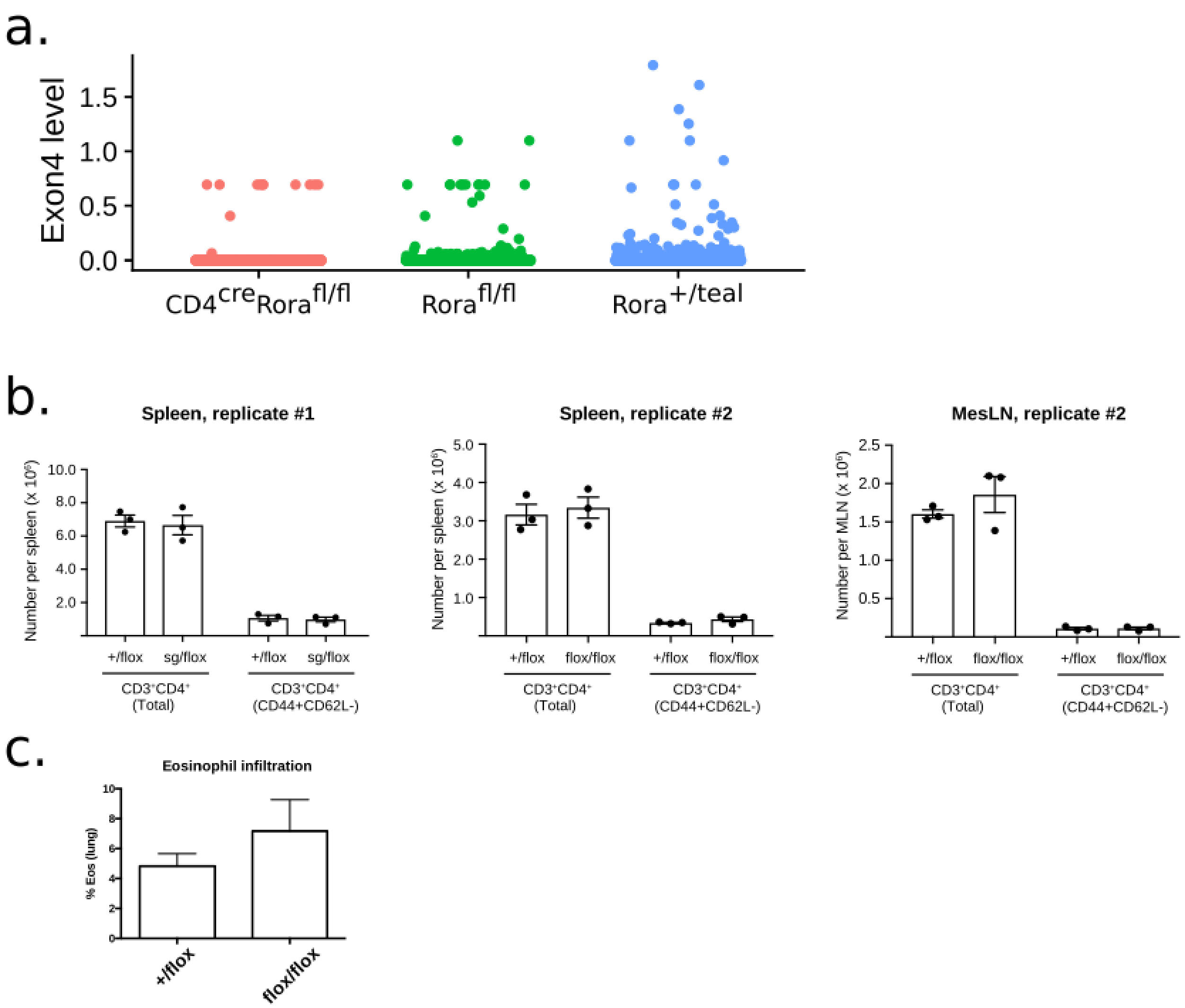

S4 Fig. (a): Usage of exon 4 in *Rora* KO (red) *vs* control mouse (green and blue), based on scRNA-seq data. The relative exon usage is calculated as Log[(exon4_reads+1)/(rora_reads+1)]. Exon 4 is conditionally removed in the KO mouse. Note that due to intrinsic noise in scRNA-seq data, the presence of exon 4 cannot be assessed for all cells. **(b)** CD4+ T cell count does appear affected in Cd4^{Cre}*Rora*$^{fl/fl}$ KO vs control under non-infected conditions. **(c)** Eosinophil infiltration level in KO vs control, as indicated by a manual scoring; toward the direction expected for higher inflammation, but not significant.

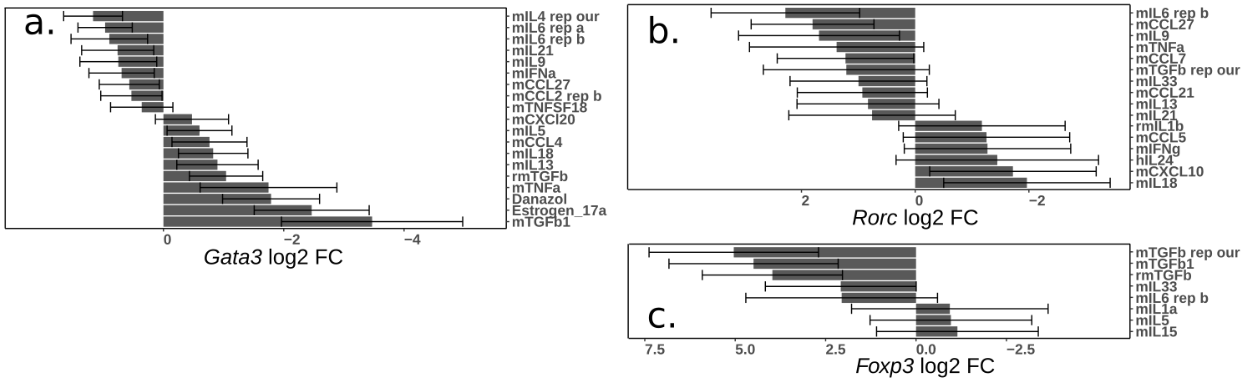

S5 Fig: Quality control of the database of cytokine-treated CD4+ T helper cells. **(a)** Regulators of Th2 cells. **(b)** Regulators of Th17 cells. **(c)** Regulators of Treg cells.

References

1. T. Lönnberg, V. Svensson, K. R. James, D. Fernandez-Ruiz, I. Sebina, R. Montandon, M. S. F. Soon, L. G. Fogg, A. S. Nair, U. Liligeto, M. J. T. Stubbington, L.-H. Ly, F. O. Bagger, M. Zwiessele, N. D. Lawrence, F. Souza-Fonseca-Guimaraes, P. T. Bunn, C. R. Engwerda, W. R. Heath, O. Billker, O. Stegle, A. Haque, S. A. Teichmann, Single-cell RNA-seq and computational analysis using temporal mixture modelling resolves Th1/Tfh fate bifurcation in malaria. *Sci Immunol*. **2** (2017), doi:10.1126/sciimmunol.aal2192.

2. R. J. Miragaia, T. Gomes, A. Chomka, L. Jardine, A. Riedel, A. N. Hegazy, N. Whibley, A. Tucci, X. Chen, I. Lindeman, G. Emerton, T. Krausgruber, J. Shields, M. Haniffa, F. Powrie, S. A. Teichmann, Single-Cell Transcriptomics of Regulatory T Cells Reveals Trajectories of Tissue Adaptation. *Immunity*. **50**, 493–504.e7 (2019).

3. S. Davidson, M. Efremova, A. Riedel, B. Mahata, J. Pramanik, J. Huuhtanen, G. Kar, R. Vento-Tormo, T. Hagai, X. Chen, M. A. Haniffa, J. D. Shields, S. A. Teichmann, Single-Cell RNA Sequencing Reveals a Dynamic Stromal Niche That Supports Tumor Growth. *Cell Rep.* **31**, 107628 (2020).
